# Accuracy of Acetabular Cup Placement During Total Hip Arthroplasty in Supine Position Using a Pelvic Rotation Correction Device

**DOI:** 10.1016/j.artd.2022.04.004

**Published:** 2022-05-23

**Authors:** Satoshi Nakasone, Mika Takaesu, Masato Ishihara, Masamichi Onaga, Takahiro Igei, Yoshihide Miyata, Kotaro Nishida

**Affiliations:** a Department of Orthopedic Surgery, Graduate School of Medicine, University of the Ryukyus, Okinawa, Japan; b Department of Orthopedic Surgery, Chubu Tokushukai Hospital, Okinawa, Japan; c Department of Orthopedic Surgery, Nakagami Hospital, Okinawa, Japan

**Keywords:** Cup placement assist device, Functional pelvic plane, Total hip arthroplasty, Direct anterior approach, Supine position, Pelvic rotation

## Abstract

**Background:**

Accurate cup placement during total hip arthroplasty (THA) is difficult because the intraoperative pelvic position changes even in supine patient position. We developed a device known as HipPointer; it corrects pelvic rotation and creates a functional pelvic plane as a reference. The aim of this study was to determine the device placement accuracy and investigate causes of error.

**Material and methods:**

HipPointer was used for cup placement in 353 hips of 308 patients who underwent direct-anterior-approach THA in supine position. The mean age at surgery and body mass index were 63.9 (17-90) years and 24.9 (16.6-42.0) kg/m^2^, respectively. The mean observation period was 40.5 (12-73) months. To investigate the accuracy of HipPointer, preoperative planning and postoperative cup placement angles relative to the functional pelvic plane were evaluated using a three-dimensional analysis software, and absolute errors were determined.

**Results:**

The means ± standard deviations of radiographic inclination (RI) and radiographic anteversion (RA) were 40.2 ± 3.0° and 15.8 ± 3.6°, respectively. The absolute errors of RI and RA were 2.2 ± 2.0° and 2.7 ± 2.3°, respectively. The ratio of the cup placement angle for which both RI and RA are ≤10° in the target zone was 99% (350/353 hips), and the ratio of the absolute errors for which both RI and RA are ≤5° was 80.4% (284/353 hips).

**Conclusions:**

HipPointer is simple in structure, easy to use, and useful for direct-anterior-approach THA in supine position. It provides good cup placement accuracy.

## Introduction

Total hip arthroplasty (THA) is a surgical procedure that relieves pain, increases the range of motion of the hip joint, and improves the quality of life of patients [[1], [2], [3], [4]]. Accurate cup placement during THA is important because it affects the risk of postoperative dislocation and the long-term results of implants [[5], [6], [7], [8]]. In recent times, the intermuscular approach of minimally invasive surgery has become common [[9], [10], [11]]. THA approaches are broadly categorized as direct-anterior [12], anterolateral [11], or posterior [13], and the advantages and disadvantages of each approach are reported in previous studies [[14], [15], [16], [17]].

The advantages of direct-anterior-approach THA (DAA-THA) performed in supine position include preservation of muscles and tendons, facilitation of acetabular exposure, and ease of cup placement determination intraoperatively using fluoroscopy [18,19]. However, leverage force applied for acetabular exposure using retractors causes intraoperative lateral pelvic rotation [[20], [21], [22]], which shows that the cup placement angle changes depending on the position of the fluoroscope [18,19]. Therefore, for accurate cup placement, it is important to carefully evaluate pelvic rotation intraoperatively.

An anatomical pelvic plane that passes through the bilateral anterior superior iliac spines (ASISs) and the pubic symphysis is commonly used as a three-dimensional (3D) pelvic coordinate system for computer navigation. Furthermore, the functional pelvic plane (FPP), a horizontal plane that runs through the bilateral ASISs and is parallel to the computed tomography (CT) imaging table in supine position, is often used as the reference plane for 3D preoperative planning of cup inclination and anteversion [23,24]. In addition, the FPP is the plane of the imaging table for simple radiography in routine clinical practice; hence, the advantage of FPP as the reference plane is easily recognized by orthopedic surgeons.

In some previous studies on intraoperative assistance devices for cup placement, pelvic position was tracked intraoperatively in supine or lateral position [[25], [26]]. These studies reported good accuracy of the devices, but with some outliers. With assistance devices, it may be difficult to track pelvic position changes that occur during the surgical procedure. Therefore, we hypothesized that cup placement accuracy can be maintained if pelvic position changes are corrected such that the pelvis is parallel to the surgical bed. We designed a simple device that assists with intraoperative cup placement and can be used to correct pelvic rotation intraoperatively. The device is called HipPointer, and it uses horizontal levels to create FPP as a reference. The aim of this study was to evaluate the accuracy of cup placement of HipPointer and to investigate errors in cup placement accuracy.

## Material and methods

This retrospective study was approved by the institutional review board to which authors belong (study approval number: 1839). In this study, we evaluated 353 hips of 308 patients (93 male and 215 female patients) who underwent cup placement during DAA-THA in supine position using HipPointer between June 2016 and September 2020. Their mean age at surgery and mean body mass index (BMI) were 63.9 (range: 17-90) years and 24.9 (range: 16.6-42.0) kg/m^2^, respectively. The mean observation period was 40.5 (range: 12-73) months. The pathology was osteoarthritis in 244 hips, femoral head necrosis in 80 hips, rheumatoid arthritis in 15 hips, rapidly destructive coxarthrosis in 8 hips, and posttrauma osteoarthritis in 6 hips (Table 1); 2 hips were excluded from this study. In one of the excluded hips, a change in cup angle of 20° was observed in anteversion, and a greater change was observed intraoperatively after eccentric screw fixation (inclination angle: 48°, anteversion: 35°). The other hip was treated for purulent hip arthritis in childhood, resulting in pelvic asymmetry and different heights of ASISs.

**Table 1:** Patient demographics, 308 cases with 353 hips.

Male:Female	93:215
Mean age (range)	63.9 (17 to 90)
Mean BMI (range)	24.9 (16.6 to 42.0)
Mean follow-up period	40.5 (12 to 73) mo
Pelvic tilt	2.4° (−27.7 to 19.6)
Disorder	
Osteoarthritis (OA)	244 hips
Osteonecrosis	80 hips
Rheumatoid arthritis	15 hips
RDC	8 hips
Posttrauma OA	6 hips

RDC, rapidly destructive coxarthropathy.

All the patients underwent preoperative CT, and 3D preoperative planning was performed using a 3D templating software program (ZedHip; Lexi Co., Ltd., Tokyo, Japan) with market-available computer-aided design data of the prosthesis. First, CT data were imported into the software, and FPP was created using digitized bilateral ASISs parallel to the CT table. A cup size that fits the anteroposterior diameter of the acetabulum was selected, and cup placement with a cup-center-edge angle ≥10° was planned. The cup placement angles, which were defined radiographically and relative to the FPP, were determined as follows: Inclination was fixed at 40° (ie, radiographic inclination [RI]: 40), and anteversion was determined as 10°, 15°, or 20° (ie, radiographic anteversion [RA]: 10°, 15°, or 20°) according to pelvic posterior tilting in the standing position, femoral head anteversion, and acetabular anterior wall coverage, to avoid iliopsoas irritation by cup protrusion.

**Figure 1:**
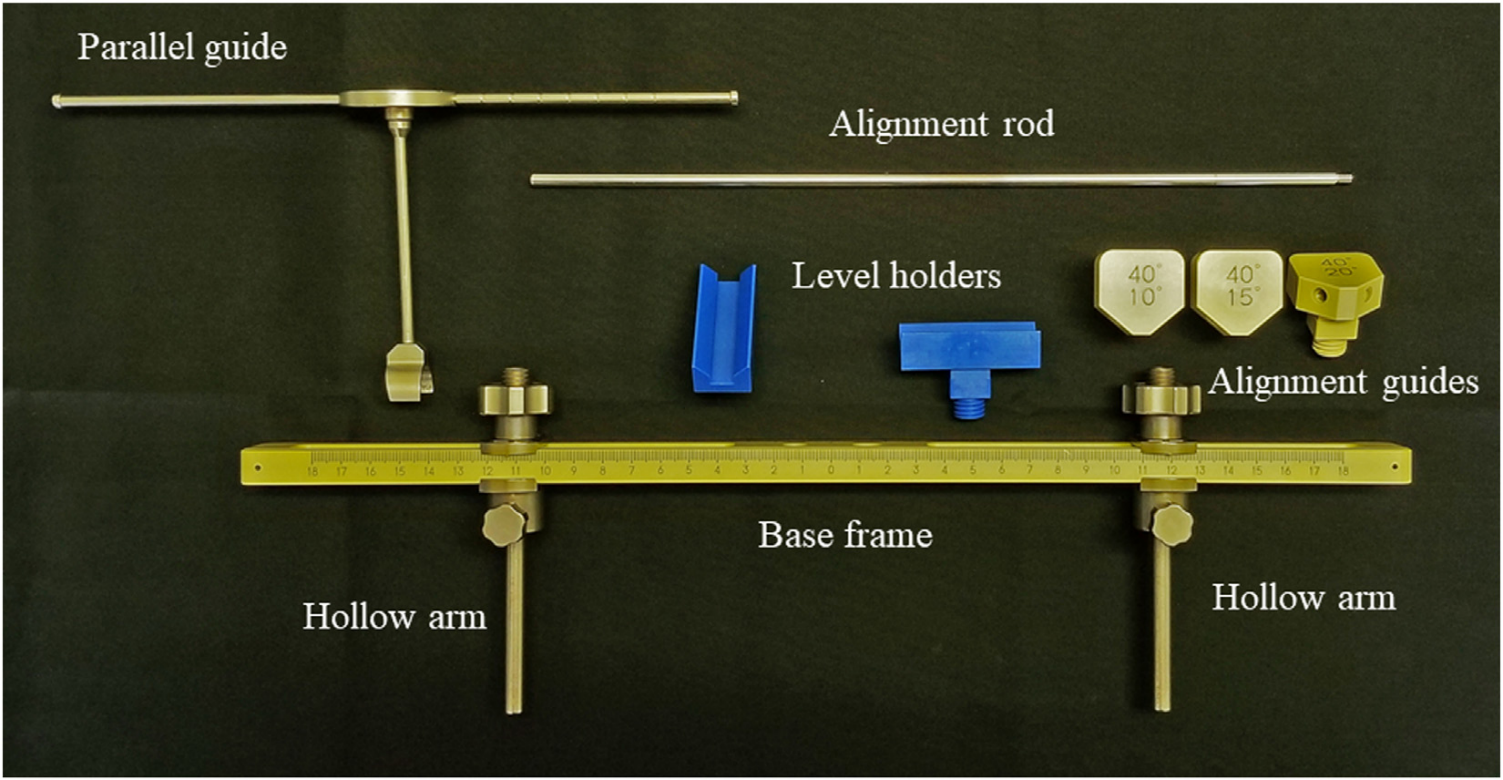
Structure of HipPointer; HipPointer consists of a base frame, 2 hollow arms attached to the base frame, 2 level holders, 3 alignment guides, an alignment rod, and a parallel guide to which is attached a cup impactor.

**Figure 2:**
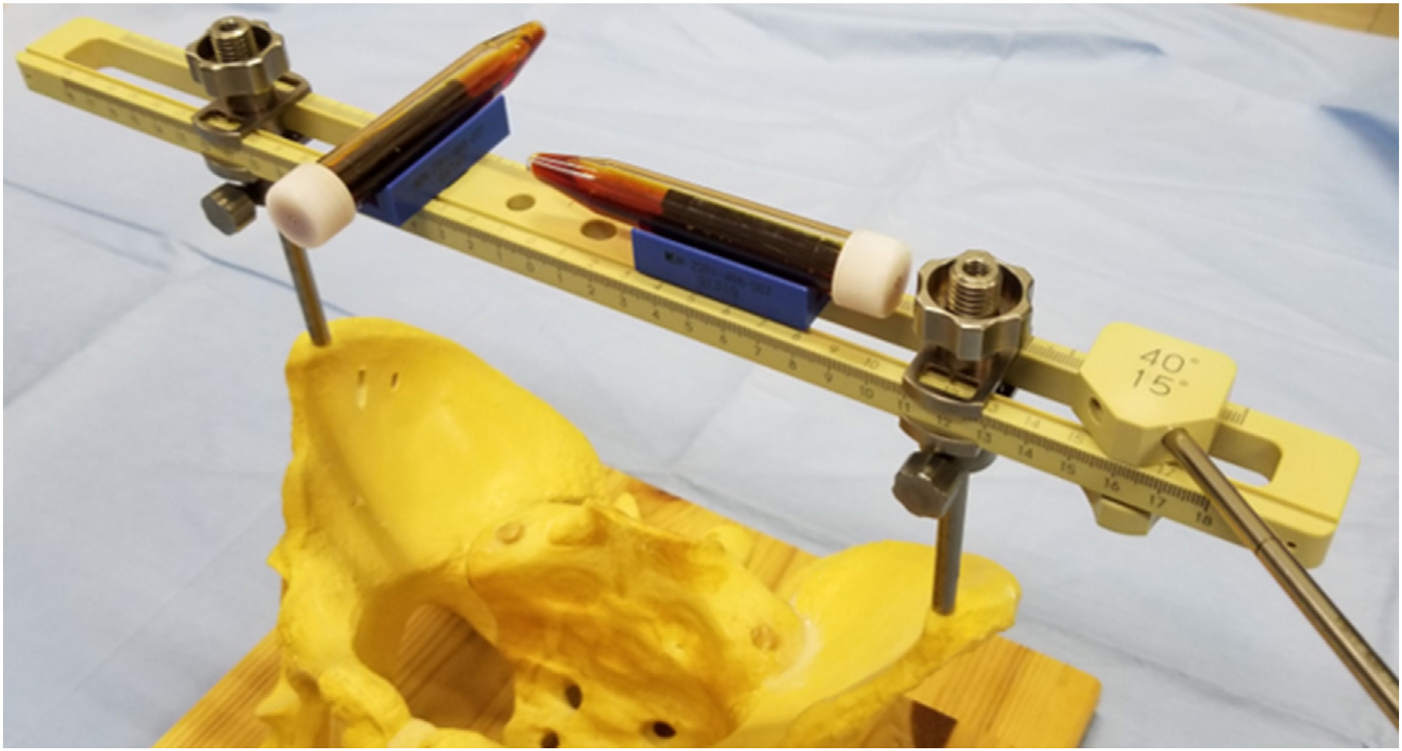
Assembly of HipPointer components; an alignment guide with an attached alignment rod is placed on the base frame, and level holders are attached to the base frame. Sterile tubes that contain iodine diluted with saline are placed on the level holders to confirm that the base frame is on a horizontal level.

**Figure 3:**
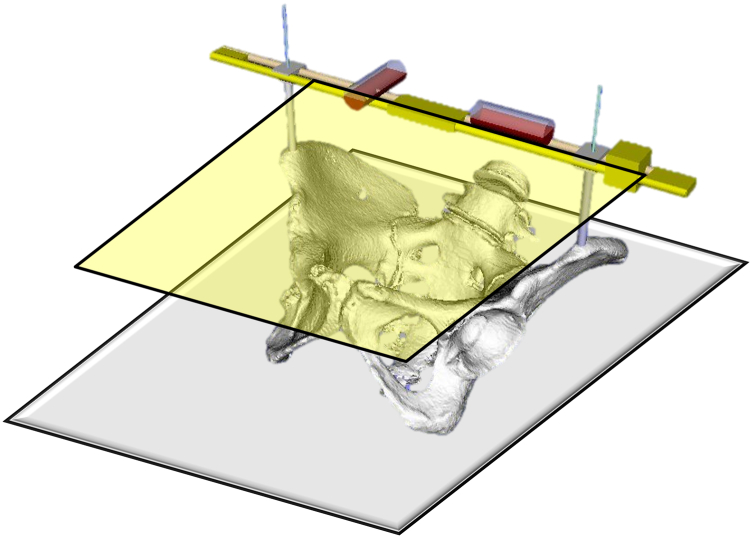
Intraoperative recreation of the FPP. The FPP is recreated by leveling the left and right ASISs and the base frame horizontally along the sagittal plane using HipPointe.

HipPointer (medical approval number: 13B1X00213MD0012), which is made by Japan MDM Inc. (Tokyo, Japan), consists of a long and narrow base frame made from polyether ether ketone, which is radiolucent and light, and 2 hollow stainless steel arms that can be adjusted to match the width between the left and right ASISs. The device can be sterilized in an autoclave (Fig. 1). Regarding accessories, level holders and an alignment guide are attached to the base frame when the device is in use. Physiological saline colored with Popiyodon Solution 10% (povidone iodine, Yoshida Pharmaceutical, Tokyo, Japan), which is available in any operating room, is poured into sterile tubes and used to determine the horizontal level. Two level holders are attached orthogonally to the base frame and used to indicate when inclination matches the horizontal plane (Fig. 2). HipPointer has 2 roles to create FPP intraoperatively. The level holder attached parallel to the base frame is used to ensure that the pelvis is parallel to the horizontal plane, while the other level holder attached orthogonally to the device is used to ensure the base frame is parallel to the operating table (Fig. 3). There are 3 alignment guides, each with a fixed inclination of 40° and anteversion of 10°, 15°, or 20°, as defined radiographically (Fig. 1). Each alignment guide has separate holes on the left and right, and the alignment rod can be screwed into any of the holes.

**Figure 4:**
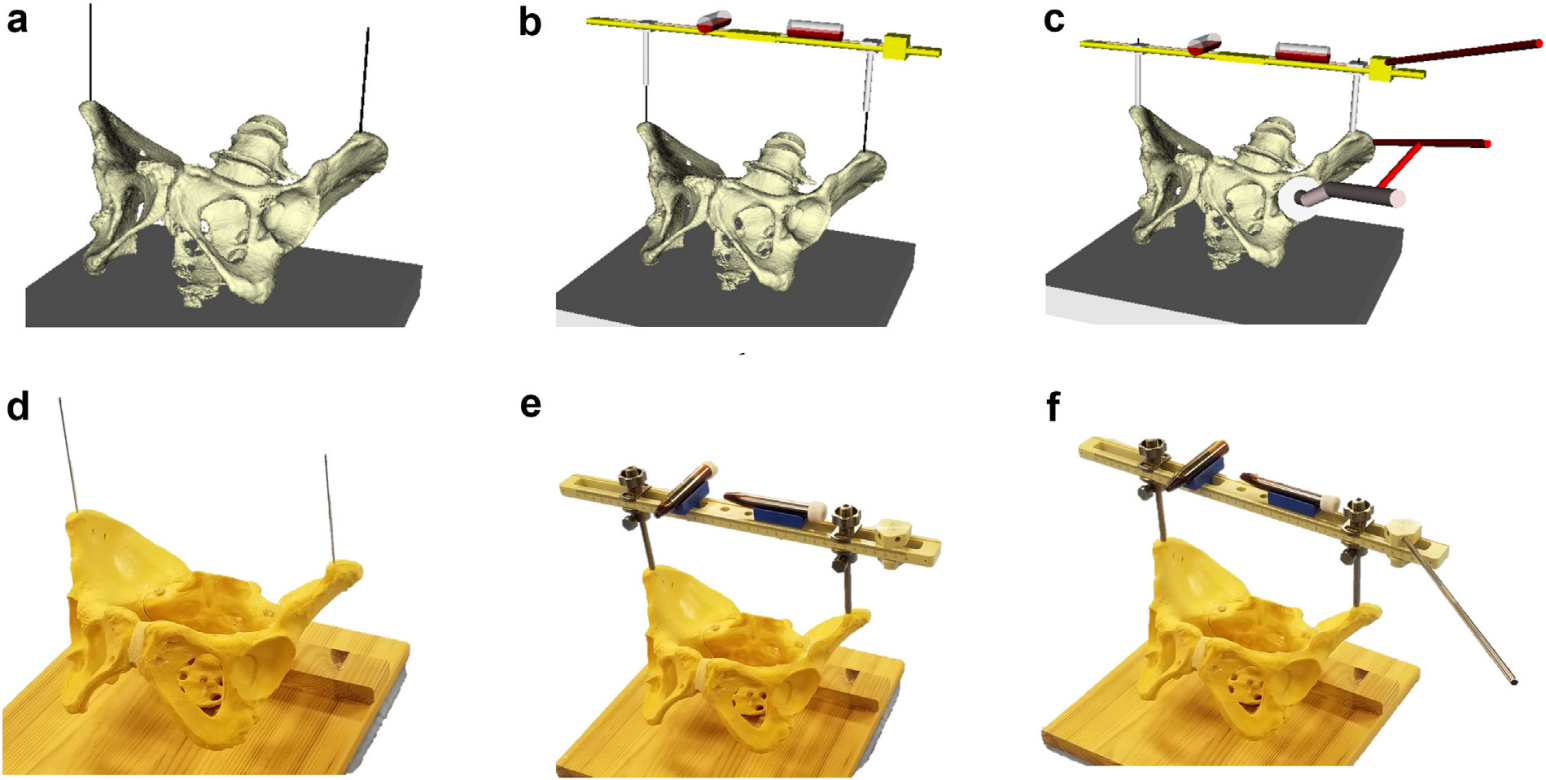
Mounting the HipPointer (computer model and plastic model); K-wires are inserted in the left and right ASISs (a, d) and passed through the hollow arms to mount the device on the pelvis. Level holders are attached to the base frame (b, e). The procedure takes approximately 1 minute (c, f).

**Figure 5:**
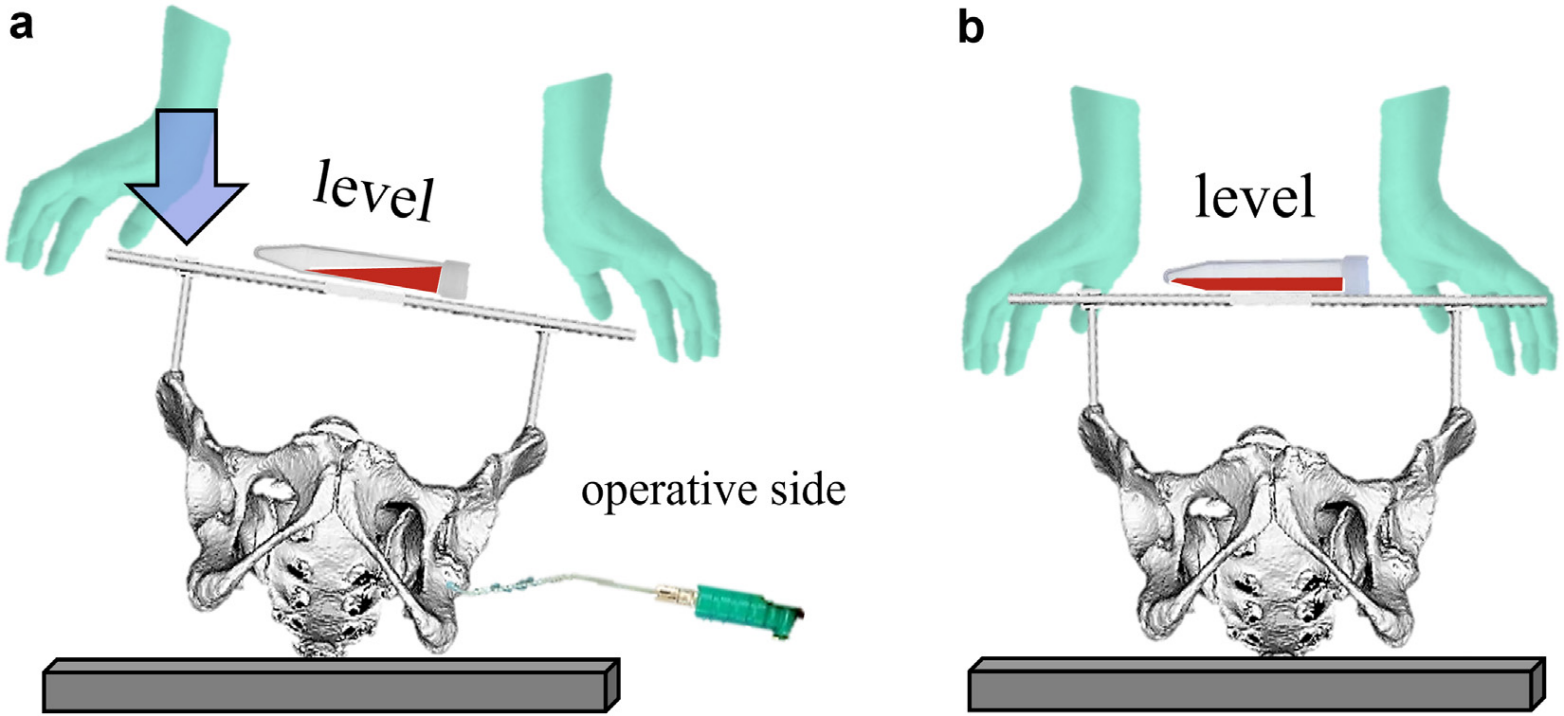
Correction of pelvic rotation using HipPointer; during surgery, leverage forces applied through retractors cause pelvic tilt (a). After the retractors are removed, an assistant returns the pelvis to the horizontal position by adjusting the left and right ASISs (b).

**Figure 6:**
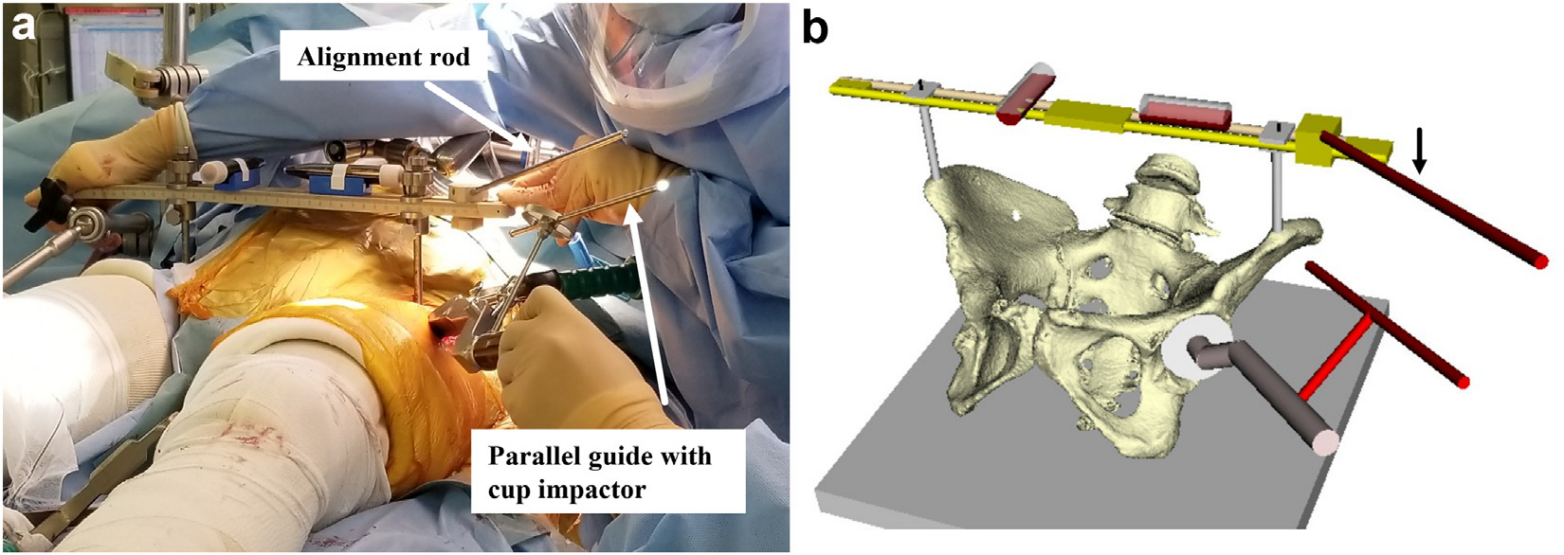
Intraoperative photograph and computer model; after cup placement, the retractors around the acetabulum are removed. An assistant corrects the pelvic rotation and recreates the FPP using HipPointer. The cup is press-fitted and fixed with the alignment rod and parallel guide.

**Figure 7:**
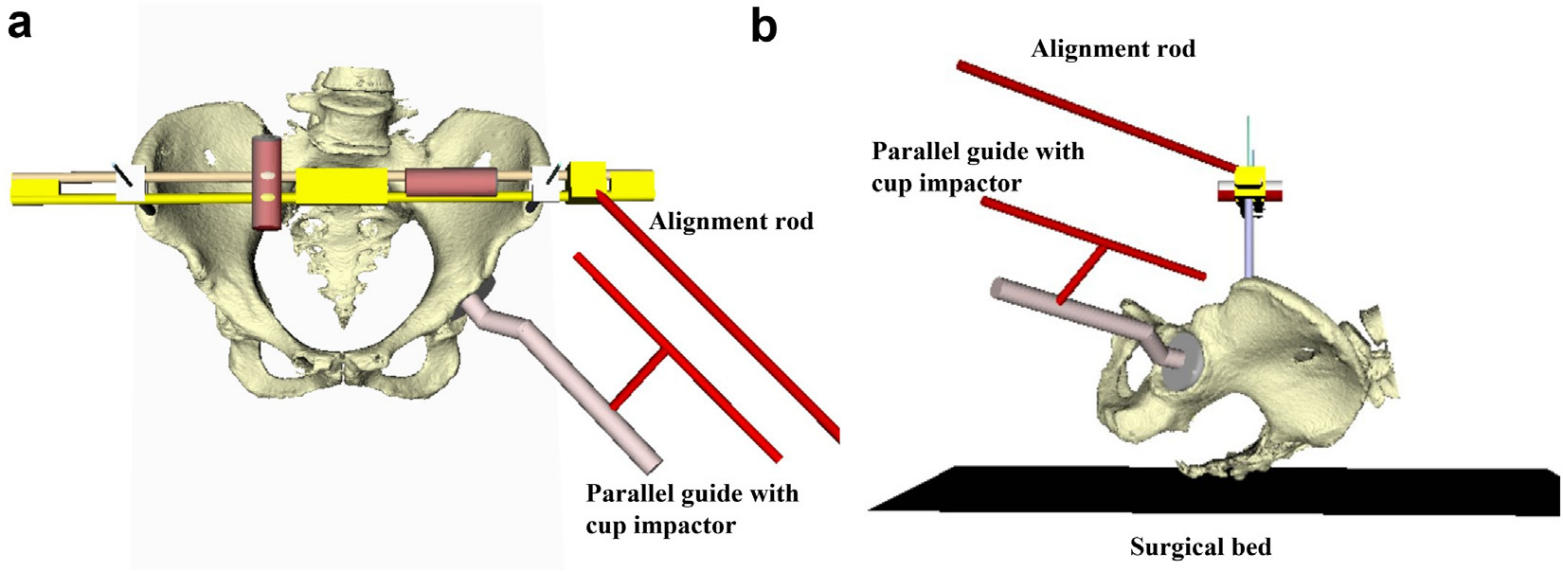
Key to accurate cup placement. The key to accurate alignment is checking the alignment from directly above (a) and from the side (b) rather than from the surgeon’s perspective alone.

All the patients underwent DAA-THA in supine position. The ASISs were examined for touch after general anesthesia. For those with obesity, the belly fat was pulled up and secured with tape before surgery to touch the ASIS during surgery. The skin was incised on the anterolateral aspect of the hip joint, the tensor fasciae latae muscle was retracted laterally, and the deep layer was exposed. A V-shaped incision was made on the joint capsule, and the incised joint capsule was reflected. After femoral neck osteotomy and acetabular exposure, we started HipPointer placement. First, 2.0-mm Kirschner wires (K-wires) were inserted to mark the bilateral ASISs. A 2.0-mm K-wire was inserted into the skin over the ASIS, the width of the ASIS was checked with the tip of the pin, and the pin was inserted centrally. The K-wires were passed through the hollow arms, which were matched to the width between the bilateral ASISs, and HipPointer was mounted on the left and right ASISs. Next, the alignment guide that reflects the preoperative plan was placed on the base frame, and the 2 orthogonal level holders were attached. This procedure took approximately one minute (Fig. 4). With the HipPointer mounted, the acetabulum was reamed progressively with 1-mm underreaming. The cup was inserted into the acetabulum, and then the retractors were removed to eliminate the force applied to the acetabulum. Finally, an assistant pushed on the HipPointer and, through the hollow arms, adjusted the rotation of the axial plane of the bilateral ASISs and pelvis to match the horizontal plane using 1 level holder and adjusted the sagittal plane of the base frame using the other orthogonal level holder (Fig. 5). Thus, the FPP, which passes through the left and right ASISs and is parallel to the plane of the operating table, was recreated using the orthogonally mounted level holders to adjust the sagittal plane of the base frame (Fig. 3). The parallel guide is attached to the cup impactor handle, and the cup was fixed in place so that the parallel guide is parallel to the alignment rod attached to the alignment guide (Fig. 6). The key to accurate cup placement is confirming that the guided alignment rod and parallel guide with the cup impactor are parallel when viewed directly from above and the side rather than from the surgeon’s point of view alone (Fig. 7). After press-fit fixation, the quality of cup fixation was confirmed by the synchronized movement of the pelvis. Moreover, the cup placement angle and gap between the cup and acetabulum were confirmed by an image intensifier. To obtain secure stability of the cup, 1 or 2 fixation screws were placed into the superior or posterior cup holes. The HipPointer and 2.0-mm K-wires were removed immediately after cup fixation. The procedure then shifted to the femoral aspect. The femur was prepared, and the stem was placed. After the surgical wound is closed, the insertion wounds from the 2.0-mm K-wires were covered with gauze alone.

Full weight-bearing was allowed for all the patients immediately after the surgery. Clinical assessments were performed preoperatively and at the most recent follow-up using the Japanese Orthopaedic Association (JOA) hip score [27]. The JOA hip score is the total score of 4 subscales for pain, range of movement, gait, and activities of daily living, with subscale score ranges of 0 to 40, 0 to 20, 0 to 20, and 0 to 20, respectively. The total score is a maximum 100 points for asymptomatic patients, and a strong correlation was observed between JOA hip score and Harris hip score [27,28].

All patients underwent CT scan 1 or 2 weeks after surgery, and the same 3D templating software was used to analyze cup placement angle. After CT data were imported, the computer-aided design model was superimposed on the implanted cup in the axial, sagittal, and coronal views. The cup placement angles relative to the FPP were automatically calculated. To measure the preoperative and postoperative pelvic sagittal tilts, the coordinate system of the pelvis was determined on each CT image using anatomical landmarks. Pelvic sagittal tilt was defined as the angle between the anatomical pelvic plane and the CT table. To determine the accuracy of cup placement using HipPointer, the absolute errors of the radiographically defined preoperative and postoperative cup inclination angle (ie, RI) and anteversion angle (ie, RA) relative to the FPP were evaluated. In addition, the correlation between error and patient demographics was investigated.

Statistical analyses were performed using JMP software version 15.0 (SAS Institute, Cary, NC). We compared continuous and nominal variables between groups using Mann-Whitney U test and chi-square test, respectively. Spearman correlation coefficient test was used to determine the correlation between error and patient demographics. *P* values less than 0.05 were considered significant. To determine cup placement accuracy, we assessed intraobserver and interobserver reliabilities for RI and RA, which were both 0.99.

## Results

The mean JOA hip score improved from 65 preoperatively to 90 postoperatively. A greater trochanteric fracture was observed intraoperatively in 2 patients. It was postoperatively observed that 1 patient had sciatic nerve palsy, anterior dislocation, and posterior dislocation, and periprosthetic femoral fractures were observed in 4 patients. Femoral nerve palsy or deep infection was not observed in any patient. There were no complications (eg, dermopathy due to pressure from the hollow arms and infection of the K-wire insertion sites) resulting from HipPointer use.

**Figure 8:**
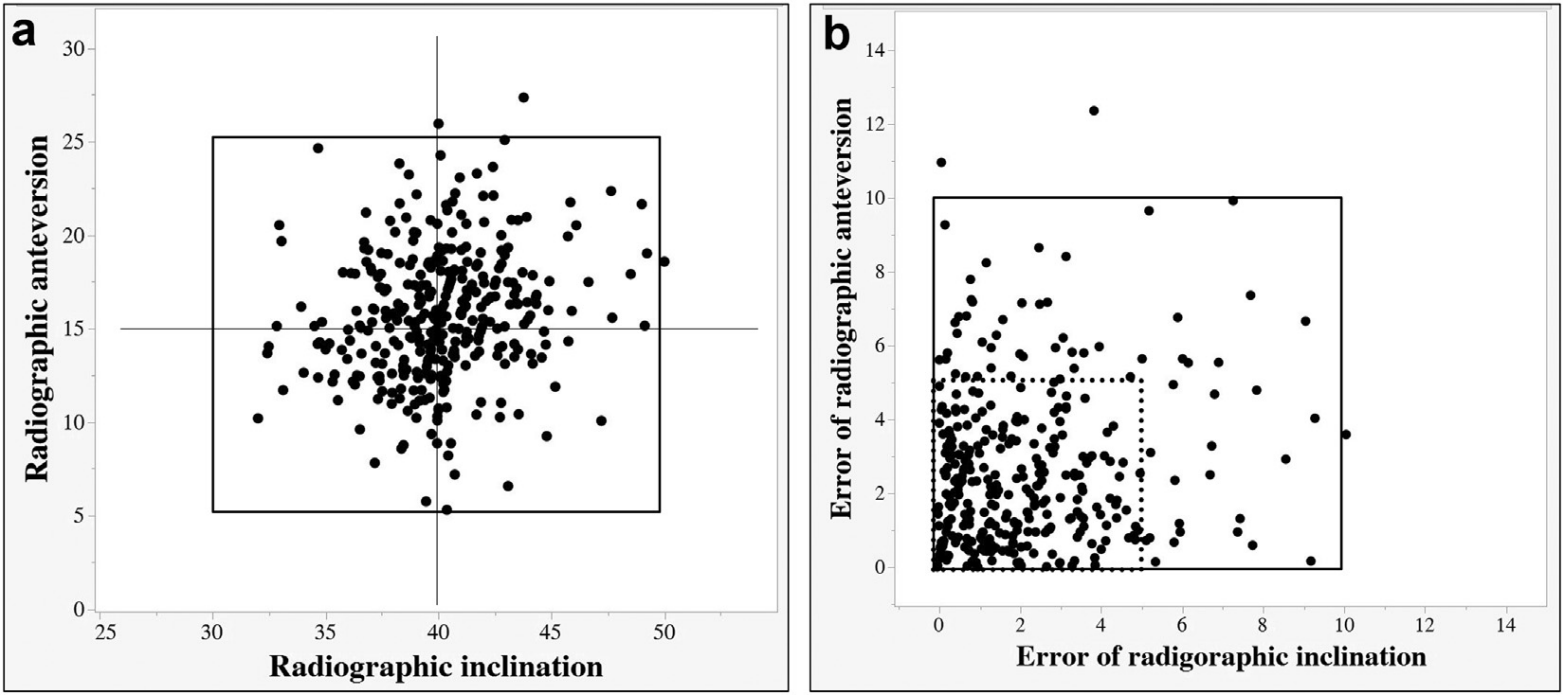
Cup placement angle and absolute errors. The ratio of cup placement angle for which both RI and RA are ≤10*°* in the target zone (line square) is 99.1% (350/353 hips) (a), and the ratio of absolute errors for which both RI and RA are ≤5° (dot square) is 80.4% (284/353 hips) (b).

The means ± standard deviations of RI and RA were 40.4° ± 3.0° and 15.8° ± 3.6°, respectively (Table 2). The absolute error of cup placement was 2.2° ± 2.0° for RI and 2.7° ± 2.3° for RA (Table 2). The ratio of the hips in the target zone for which both errors of RI and RA are ≤10° (with inclination angle ranging from 30° to 50° and anteversion angle ranging from 5° to 25°) was 99% (350/353 hips) [29], and the ratio in the Sugano target zone for which both errors of RI and RA are ≤5° was 80% (284/353 hips) (Fig. 8) [30]. Anterior dislocation occurred in a 37-year-old woman with femoral head necrosis, RI of 40°, and RA of 15°. Posterior dislocation occurred in a 79-year-old woman with rapidly destructive coxarthrosis due to rheumatoid arthritis, RI of 40°, and RA of 17°. The condition of these 2 women may not cause poor cup placement. No correlation was observed between BMI and errors of RI or RA (Table 3).

**Table 2:** Cup placment angle and absolute errors of RI and RA.

Variables	Value	Absolute error
RI (mean ± SD)	40.2 ± 3.0°	2.2 ± 2.0°
RA (mean ± SD)	15.8 ± 3.6°	2.7 ± 2.3°

RI, radiographic inclination, RA, radiographic anteversion.

**Table 3:** Spearman's correlation between absolute errors and patient demographics.

Patient demographics	Errors of RI	Errors of RA
Age	−0.17	−0.06
BMI	−0.01	−0.08
Pelvic tilt		
Preoperative	0.06	0.06
Postoperative	0.09	0.1
Differences	0.05	0.07

BMI, body mass index.

## Discussion

This study showed that cup placement accuracy is within 3° degrees for inclination and anteversion. The accuracy with HipPointer appears comparable to the accuracies with computer-assisted navigation and robot-assisted surgery [[31], [32], [33]]. Cup placement accuracy relates to precise tracking of pelvic position changes. While computer-assisted navigation and robot-assisted surgery can increase implantation accuracy, their use is limited by high cost of software/hardware, maintenance, and technical complexity. In contrast, with HipPointer, only 2 procedures are needed for manual creation of FPP. The surgical assistant first corrects pelvic rotation and then adjusts the frame tilt to the horizontal level. The 2.0-mm K-wires at both ASISs allow for appropriate mounting of the arm of HipPointer onto the pelvis, with no need for perpendicular insertion into the surgical bed. Therefore, it was not necessary to use computer-assisted navigation or robot-assisted surgery or to attach a reference array to the iliac crest by inserting 2 temporary 4.0-mm Schanz screws. The 2.0-mm K-wires used were removed immediately after cup placement, and after surgery, no patient had lateral femoral cutaneous nerve injury, infection, or symptoms such as pin-site pain or irritation. Thus, HipPointer can be considered less invasive and easier to use than computer-assisted navigation or robot-assisted surgery.

Our results revealed no correlation between error and patient demographics, especially BMI. Several studies reported that intraoperative pelvic movement occurs in the supine position during THA [[20], [21], [22]]. Kamenaga et al. reported a significant negative correlation between change in absolute axial rotation and BMI [21]. In the study by Okamoto et al. in which portable navigation was used in supine THA, it was reported that pelvic rotation in patients with low BMI changed during registration and after cup placement [22]. Furthermore, they reported that the forces of the posterior retractors acted downward on the anterior thigh and caused pelvic axial rotation. In this study, pelvic rotation was observed with HipPointer following acetabular exposure using retractors. Thus, HipPointer, which can correct pelvic tilt intraoperatively regardless of BMI, is a useful device for accurate cup placement in supine THA.

The advantage of HipPointer is that it can be used for patients who cannot extend the hip due to pain from femoral neck fracture and for patients with severe hip contracture caused by severe deformity. If pelvic tilt occurred before surgery due to hip pain or contracture, the FPP referenced in preoperative planning may change after surgery, and the cup placement angle may also change. However, this may be considered an advantage of HipPointer use because the FPP was recreated after releasing the joint capsule and resecting the femoral head. HipPointer uses intraoperative FPP, not preoperative FPP, as a reference.

The disadvantage of HipPointer is that it is not easy to use in patients with ASIS asymmetry. ASIS asymmetry is thought to occur in conditions associated with iliac deformation or hypoplasia (eg, pelvic fracture, unilateral congenital hip dislocation, and purulent hip arthritis during childhood). HipPointer use in patients with ASIS asymmetry requires steps such as converting to the pelvic tilt observed during CT imaging.

The study has several limitations. This was a retrospective study involving a single approach, and further studies involving other approaches and comparisons with computer navigation in the supine position are needed. Another limitation of this study is that it evaluated cup angle using radiographically defined angles relative to the FPP. Since the FPP is affected by patient posture, preoperatively planned angles relative to FPP and angles relative to the pelvis at the time of postoperative evaluation were not compared.

HipPointer has a simple structure and consists of a base frame and arms in a compact and portable set (Fig. 1). It is made of polyether ether ketone and stainless steel and can be sterilized in nearly any operating room. Levels are created by placing diluted povidone-iodine solution in sterile tubes, both of which are readily obtainable in any operating room. Although the level holders and alignment guide are analog devices that are checked visually, the simple method and concept of HipPointer use are easy to understand, even for first-time users. Due to its affordability, simple structure, and ease of use, HipPointer is an intraoperative assistance device that should be considered for accurate cup placement during supine THA in hospitals and institutions where computer-assisted navigation or robot-assisted surgery is not yet available.

## Conclusion

The accuracy of HipPointer, a cup placement assistance device that uses the FPP as a reference for intraoperative correction of pelvic axial rotation, was evaluated in 353 hips on which DAA-THA was performed. Cup placement error was 2.2 ± 2.0° for inclination and 2.7 ± 2.3° for anteversion. HipPointer is a cup placement assistance device that has a simple structure, is easy to use, and is useful for DAA-THA in supine position.

## Appendix A Supplementary Data

Conflict of Interest Statement for Igei

Conflict of Interest Statement for Nishida

Conflict of Interest Statement for Onaga

Conflict of Interest Statement for Ishihara

Conflict of Interest Statement for Takaesu

Conflict of Interest Statement for Nakasone

Conflict of Interest Statement for Miyata
